# Effect of intraperitoneally administered recombinant murine granulocyte-macrophage colony-stimulating factor (rmGM-CSF) on the cytotoxic potential of murine peritoneal cells

**DOI:** 10.1038/sj.bjc.6690016

**Published:** 1999-09-24

**Authors:** A H Klimp, J Regts, G L Scherphof, E G E de Vries, T Daemen

**Affiliations:** Department of Physiological Chemistry, Faculty of Medical Sciences, Groningen Institute for Drug Studies, A. Deusinglaan 1, Groningen, 9713 AV; Department of Medical Oncology, Faculty of Medical Sciences, Groningen Institute for Drug Studies, A. Deusinglaan 1, Groningen, 9713 AV, The Netherlands

**Keywords:** granulocyte–macrophage colony-stimulating factor, peritoneal macrophages, murine, cytotoxicity

## Abstract

We studied the effect of recombinant murine granulocyte–macrophage colony-stimulating factor(rmGM-CSF) on the cytotoxic potential of murine peritoneal cells. Mice received rmGM-CSF intraperitoneally using different dosages and injection schemes. At different time points after the last injection, mice were sacrificed, peritoneal cells isolated and their tumour cytotoxicity was determined by a cytotoxicity assay using syngeneic [methyl-^3^H]thymidine-labelled colon carcinoma cells. Also, the cytotoxic response to a subsequent in vitro stimulation with lipopolysaccharide was determined. Upon daily injection of 6000–54 000 U rmGM-CSF over a 6-day period, the number of peritoneal cells increased over ten fold with the highest rmGM-CSF dose. Increases in cell numbers was mainly due to increases in macrophage numbers. Upon injection of three doses of 3000 U rmGM-CSF per day for 3 consecutive days, the number of macrophages remained elevated for minimally 6 days. Although the peritoneal cells from rmGM-CSF-treated mice were not activated to a tumoricidal state, they could be activated to high levels of cytotoxicity with an additional in vitro stimulation of lipopolysaccharide. Resident cells isolated from control mice could be activated only to low levels of tumour cytotoxicity with lipopolysaccharide. Tumour cytotoxicity strongly correlated with nitric oxide secretion. When inhibiting nitric oxide synthase, tumour cell lysis decreased. Thus, the expanded peritoneal cell population induced by multiple injections of rmGM-CSF has a strong tumour cytotoxic potential and might provide a favourable condition for immunotherapeutic treatment of peritoneal neoplasms. © 1999 Cancer Research Campaign


British Journal of Cancer (1999) **79**(1), 89–94
© 1999 Cancer Research Campaign

### Effect of intraperitoneally administered recombinant murine granulocyte–macrophage colony-stimulating factor (rmGM-CSF) on the cytotoxic potential of murine peritoneal cells

**AH Klimp[1], J Regts[1], GL Scherphof[1], EGE de Vries[2] and T Daemen[1]**

[1]Department of Physiological Chemistry, [2]Department of Medical Oncology, Faculty of Medical Sciences, Groningen Institute for Drug Studies, University of Groningen, A. Deusinglaan 1, 9713 AV Groningen, The Netherlands

**Summary** We studied the effect of recombinant murine granulocyte–macrophage colony-stimulating factor (rmGM-CSF) on the cytotoxic potential of murine peritoneal cells. Mice received rmGM-CSF intraperitoneally using different dosages and injection schemes. At different time points after the last injection, mice were sacrificed, peritoneal cells isolated and their tumour cytotoxicity was determined by a cytotoxicity assay using syngeneic [methyl-$^{3}$H]thymidine-labelled colon carcinoma cells. Also, the cytotoxic response to a subsequent in vitro stimulation with lipopolysaccharide was determined. Upon daily injection of 6000–54 000 U rmGM-CSF over a 6-day period, the number of peritoneal cells increased over ten fold with the highest rmGM-CSF dose. Increases in cell numbers was mainly due to increases in macrophage numbers. Upon injection of three doses of 3000 U rmGM-CSF per day for 3 consecutive days, the number of macrophages remained elevated for minimally 6 days. Although the peritoneal cells from rmGM-CSF-treated mice were not activated to a tumoricidal state, they could be activated to high levels of cytotoxicity with an additional in vitro stimulation of lipopolysaccharide. Resident cells isolated from control mice could be activated only to low levels of tumour cytotoxicity with lipopolysaccharide. Tumour cytotoxicity strongly correlated with nitric oxide secretion. When inhibiting nitric oxide synthase, tumour cell lysis decreased. Thus, the expanded peritoneal cell population induced by multiple injections of rmGM-CSF has a strong tumour cytotoxic potential and might provide a favourable condition for immunotherapeutic treatment of peritoneal neoplasms.

**Keywords:** granulocyte–macrophage colony-stimulating factor; peritoneal macrophages; murine; cytotoxicity

Granulocyte–macrophage colony-stimulating factor (GM-CSF) is a 20- to 30-kDa glycoprotein. Secretion can be induced by immune activation or bacterial cell wall products in endothelial cells, fibroblasts, macrophages and T-lymphocytes. GM-CSF has a broad haemopoietic specificity and acts on neutrophils, eosinophils, monocytes, macrophages, erythroid progenitors, megakaryocyte progenitors and antigen-presenting dendritic cells. These actions include prolongation of survival (inhibition of apoptosis) of progenitor cells and, in the case of mature cells, enhancement of functional capacity (Inamura et al, 1990; Molloy et al, 1995; Nagler et al, 1996). GM-CSF administration, i.v. as well as i.p., results in an increase in circulating neutrophils, eosinophils, monocytes and all progenitor cells and in an increase in numbers and activation status of tissue macrophages (Metcalf et al, 1987; Pojda et al, 1989; Ulich et al, 1990; Selgas et al, 1996), recently reported in a human study in which GM-CSF was given i.p. to dialysis patients. They showed that intraperitoneal GM-CSF caused a marked and transient recruitment of primed macrophages into the peritoneum without causing major side-effects (Selgas et al, 1996). Therefore, intraperitoneal injections of GM-CSF could be an efficient way to activate peritoneal cells, which mainly consist of macrophages, in the immunotherapeutic treatment of peritoneal cancers. In our study, we show that newly recruited macrophages, due to GM-CSF administration i.p., have high cytotoxic capacity and are able to produce large amounts of nitric oxide when activated with lipopolysaccharide (LPS).

*Received 19 December 1997*
*Revised 10 April 1998*
*Accepted 29 April 1998*

*Correspondence to:* T Daemen

About 70–80% of the peritoneal cell population in mice consists of macrophages, the remainder mainly representing lymphocytes (Plasman and Vray, 1993; DaMatta et al, 1995). Because macrophages are able to recognize and interact with tumour cells in vivo, they play an important role in host surveillance against tumour cells (Thomas et al, 1995; Aliprantis et al, 1996; Trulson et al, 1996). In contrast to constitutive functions of macrophages, such as phagocytic removal of cell debris, the macrophage requires activation signals to lyse tumour cells (Fidler, 1992). Macrophages can be activated in vivo and in vitro by contact with micro-organisms or their products (e.g. LPS, muramyldipeptide) (Daemen et al, 1986, 1989; Fidler, 1992), and also by macrophage-activating factors such as interferon-gamma and GM-CSF (Grabstein et al, 1986; Malik et al, 1991; Dileepan et al, 1995; Verstovsek et al, 1995). Activated macrophages recognize and destroy tumour cells without harming non-tumorigenic cells. The mechanism of tumour cell recognition, which is independent of major histocompatibility complex (MHC) recognition and requires cell-to-cell contact, is not yet fully understood (Fidler, 1992; Aliprantis et al, 1996). Because activated macrophages can destroy phenotypically diverse tumour cells, including cells resistant to killing by other host defence mechanisms and chemotherapeutic drugs, activation of macrophages might be exploited in immunotherapeutic treatment of small volume cancer. In the present study, we examined the effect of i.p. injections of recombinant murine granulocyte–macrophage colony-stimulating factor (rmGM-CSF) on the tumour cytotoxic properties of peritoneal cells.

### MATERIALS AND METHODS

### Animals

Female C3HeB/FeOrl-U (C3HeB) mice (8–12 weeks), obtained from the Central Animal Laboratory of the University of Utrecht, were used for the experiments. The animals received care in accordance with the institution's guidelines. The in vivo study was approved by the faculty commission of animal studies.

### Culture media and reagents

All cells were cultured in RPMI-1640 medium supplemented with 10% heat-inactivated fetal calf serum (FCS), 2 mM L-glutamine, penicillin G (100 U $ml^{-1}$) and streptomycin (100 µg $ml^{-1}$) (all from Gibco, Paisley, UK). [Methyl-$^3$H]thymidine (specific activity: 5 mCi $mmol^{-1}$) was purchased from Amersham (Buckinghamshire, UK). DNAase (1 mg $ml^{-1}$) and tumour necrosis factor alpha (TNF-α) were from Boehringer Mannheim (Mannheim, Germany). LPS, $N^G$-monomethyl-L-arginine acetate (L-NMMA), glutaraldehyde and actinomycin D were all purchased from Sigma (St. Louis, MO, USA). Bovine serum albumin (BSA) was from Sigma (Steinheim, Germany) and rmGM-CSF was purchased from PeproTech (Rocky Hill, NJ, USA).

### rmGM-CSF

rmGM-CSF produced by *Escherichia col i*(*E. coli*) had a specific activity of $10^7$ U $mg^{-1}$, as was determined by a granulocyte–macrophage colony formation assay using murine bone marrow cells. One unit was defined as the amount of rmGM-CSF necessary to stimulate the half-maximal number of colonies. rmGM-CSF was diluted in 0.1% BSA in phosphate-buffered saline (PBS) before administration. Mice were injected i.p. at the indicated times with 3000 U rmGM-CSF in a volume of 1 ml. When injecting more than once daily, an injection volume of 0.5 ml was used. Control mice were injected once daily for 3 consecutive days with 1.0 ml of 0.1% BSA in PBS.

### Peritoneal cell isolation

Mice were sacrificed by cervical dislocation. Peritoneal cells were harvested by peritoneal lavage of the peritoneal cavity with 4 ml of ice-cold RPMI-1640 medium without FCS. Cells were centrifuged at 350 ***g*** for 10 min, then the pellet was resuspended in 1 ml culture medium and cells were counted.

### Cytospots

Peritoneal cells were diluted in culture medium with 30% FCS to a concentration of $0.25 \times 10^6$ cells $ml^{-1}$. Per cytospot, $5 \times 10^4$ cells were centrifuged for 4 min at 80 ***g*** in a cytospin centrifuge (Shandon, Cheshire, UK). The cytospots were stained with May–Grünwald Giemsa staining.

### Cytotoxicity assay

Tumoricidal activity of macrophages was measured as described by Daemen et al (1986) for liver macrophages. Briefly, peritoneal cells were cultured in a 96-well plate with $0.25 \times 10^6$ cells per well. Cells were stimulated in vitro with LPS (100 ng $ml^{-1}$). In control wells, only medium was added. C26 colon carcinoma cells, syngeneic with BALB/c mice, in exponential growth phase were radiolabelled by a 20-h incubation period in medium containing 0.2 µCi of [$^3$H]dThd $ml^{-1}$. The cells were then washed free from radioisotope and cold-pulsed by incubation in fresh medium for 3–4 h to deplete cytoplasmic pools of [$^3$H]dThd and minimize spontaneous release of label. Subsequently, the cells were washed twice with PBS, 37°C, to remove unbound radiolabel, and harvested by short trypsinization (0.05% Difco trypsin–0.2% EDTA for 1 min). After four washing steps (three times with PBS, once with medium), the cells were resuspended in medium at a concentration of $5 \times 10^4$ cells $ml^{-1}$. Two hours after the addition of LPS, $10^4$ [$^3$H]dThd-labelled target cells were added per well. Radiolabelled target cells were also plated alone, as an additional control. Forty-eight hours after the addition of tumour cells, the supernatants were collected, and the radioactivity was measured in a liquid scintillation counter. Total d.p.m. added per well was determined by measuring radioactivity of $10^4$ tumour cells in 200 µl of medium mixed with 25 µl of 10% sodium dodecylsulphate (SDS). Specific cytolysis was calculated as follows:

$$\% \text{ cytolysis} = 100 \times (a-b)/(c-b)\%$$

in which *a* is d.p.m. released in the supernatant of tumour cells cultured with test macrophages, *b* is d.p.m. released in the supernatants of tumour cells cultured with control macrophages and *c* is the total d.p.m. added per well.

### Nitric oxide assay

Nitric oxide release by the cells was measured by adding 100 µl of Griess reagent (1.0% sulphanilamide, 2.5% phosphoric acid, 0.1% *N*-naphthyl-ethylene-diamine) to 100 µl of culture supernatant. After a 10-min incubation period at room temperature, optical density of the solutions was measured at 550 nm using a microtitre plate reader. A standard curve with sodium nitrite was used for calculating the final nitric oxide concentration in the culture supernatants.

### Nitric oxide synthase inhibition

Nitric oxide synthase was inhibited by adding 0.1 µM L-NMMA dissolved in PBS, to $0.25 \times 10^6$ cells cultured in 96-well flat-bottom microtitre plates in the absence or presence of LPS (100 ng $ml^{-1}$).

### TNF-α assay

TNF-α secretion was measured by an L929 assay. Briefly, $4 \times 10^4$ L929 cells in 100 µl culture medium were added to 100 µl of twofold dilutions of the culture supernatants in a flat-bottomed 96-well plate. To increase the TNF-α sensitivity of the L929 cells, 2 µl of actinomycin D (1 mg $ml^{-1}$) per $4 \times 10^5$ cells was added. After a 18–20 h incubation period, the cells were fixed with 25 µl of glutaraldehyde (25% v/v solution). After 15 min incubation, the supernatant was removed and the cells were washed with tap water and stained with 0.05% methylene blue for 20 min. Subsequently,

*© Cancer Research Campaign 1999*

the cells were washed several times and the methylene blue was extracted from the cells by adding 200 µl of 0.33 M hydrochloric acid per well. The optical density was measured at 620 nm in a microtitre plate reader. A standard curve with recombinant murine TNF-α was used to determine TNF-α concentrations in the culture supernatants. One unit of TNF-α was defined as the reciprocal dilution factor of a sample causing 50% lysis of L929 cells.

### RESULTS

### Effect of rmGM-CSF administration i.p. on cell peritoneal cell number

In Table 1, the effect of different doses of rmGM-CSF (one or three daily injections with 3000 U) and time schedules (2–6 consecutive days) on the peritoneal cell number, determined 24 h after the last injection, is shown. The number of cells increased more than tenfold with the highest dose of rmGM-CSF tested, i.e. 54 000 U rmGM-CSF totally injected. In mice injected with a single dose of 3000 U rmGM-CSF per day for 3–6 consecutive days, the cell numbers increased significantly compared with the control group. The increase in the number of cells was mainly the result of an increase in the numbers of macrophages and, to a lesser extent, neutrophilic granulocytes. Besides the change in cell number, the cell size was influenced by rmGM-CSF treatment. Peritoneal macrophages isolated from rmGM-CSF-treated animals were larger and contained more vacuoles than peritoneal cells isolated from control animals (not shown).

To determine the duration of the increase in the number of peritoneal cells upon administration of rmGM-CSF, mice were injected for 3 consecutive days with one daily injection of 3000 U rmGM-CSF. Twenty-four hours after the last rmGM-CSF injection, the number of cells showed a two- to threefold increase. The number of macrophages increased more than twofold and remained at this level for at least 6 days (Figure 1). The increase in the number of neutrophilic granulocytes was only apparent during the first 3 days and had almost disappeared after 6 days. Although the number of cells increased, we observed no increase in the number of mitotic cells (not shown).

### Cytotoxic capacity of peritoneal cells after rmGM-CSF administration

To determine whether rmGM-CSF treatment influences the tumour cytotoxicity of the peritoneal cells, the cells were isolated and co-cultured in vitro with C26 colon carcinoma cells as target cells. In addition, LPS was added 2 h before and during the entire co-culture. As shown in Figure 2, peritoneal cells isolated from control mice were not cytotoxic; they could, however, be stimulated with LPS to a level of maximally 38% of tumour cell killing.

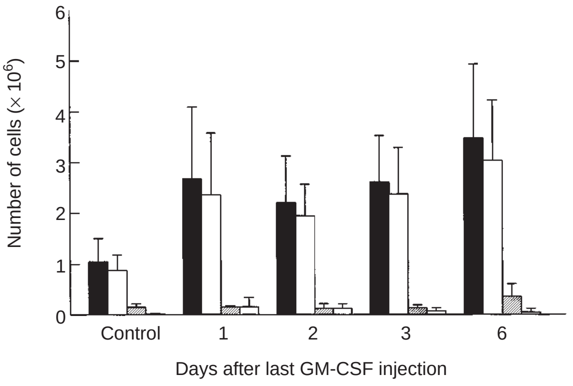

**Figure 1** Mice were treated with three daily injections of 3000 U of rmGM-CSF for 3 consecutive days. On 1, 2, 3 and 6 days after the last injection, peritoneal cells were harvested, counted and characterized. The figure shows the total number of cells (■) and the number of macrophages (□), lymphocytes (■) and neutrophilic granulocytes (□) at the different time points (±s.d., $n \geq 3$)

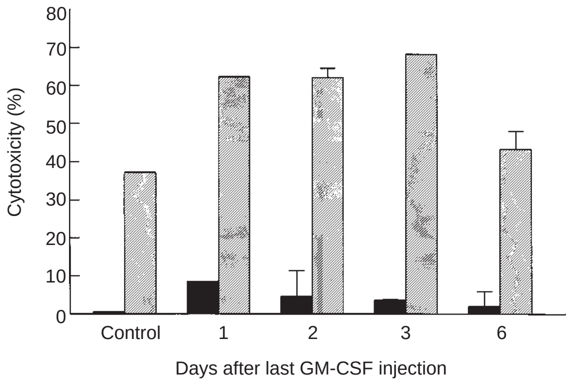

**Figure 2** Peritoneal cells were harvested from mice injected with 0.1% BSA in PBS (control group, $n = 4$) or 3000 U of rmGM-CSF ($n = 4$) for 3 consecutive days and were cultured in vitro in the absence (■) or presence of 100 ng ml$^{-1}$ LPS (▨). Cytotoxicity was determined on day 1, 2, 3 or 6 as described in the Methods and materials section. Standard deviations are given when cytotoxicity was determined in cultures from four separate mice. In the other cases, owing to a too low number of cells, supernatants were pooled and cytotoxicity was determined in triplicate. Standard deviations of the triplicate values were always below 10% of the mean value. Given is the percentage of cytolysis

**Table 1** Number of peritoneal cells after different doses of injections with rmGM-CSF

| Days of treatment | Doses per day | Total doses | Cell total (×10$^6$) | Macrophages (×10$^6$) | Lymphocytes (×10$^6$) | Neutrophils (×10$^6$) |
|---|---|---|---|---|---|---|
| 3 (control, $n = 9$) | 1 | 3 | 0.96 ± 0.63 | 0.64 ± 0.69 | 0.16 ± 0.31 | 0.07 ± 0.01 |
| 2 ($n = 3$) | 1 | 2 | 1.82 ± 0.43 | 1.27 ± 0.23 | 0.42 ± 0.19 | 0.10 ± 0.01 |
| 3 ($n = 3$) | 1 | 3 | 3.46 ± 0.73 | 2.95 ± 0.27 | 0.59 ± 0.03 | 0.09 ± 0.05 |
| 5 ($n = 3$) | 1 | 5 | 3.47 ± 1.59 | 2.69 ± 1.54 | 0.39 ± 0.06 | 0.22 ± 0.15 |
| 3 ($n = 3$) | 3 | 9 | 5.27 ± 1.59 | 4.38 ± 1.76 | 0.44 ± 0.18 | 0.40 ± 0.23 |
| 6 ($n = 3$) | 3 | 18 | 10.3 ± 3.54 | 8.41 ± 3.10 | 0.31 ± 0.11 | 1.64 ± 0.47 |

Mice were injected i.p. for 2–6 consecutive days with 0.1% BSA in PBS (control group) or with one or three daily injections of 3000 U of rmGM-CSF. Given is the total number of peritoneal cells and the number of macrophages, lymphocytes and neutrophilic granulocytes harvested 24 h after the last injection (means ± s.d.).

© Cancer Research Campaign 1999

Cells isolated from mice treated for 3 consecutive days with 3000 U rmGM-CSF were slightly more cytotoxic compared with cells isolated from control mice, nonetheless, the level of cytotoxicity was very low, maximally 8%. Cells isolated from mice injected with rmGM-CSF and subsequently stimulated in vitro with LPS were activated to high levels of cytotoxicity, up to 65–70% (Figure 2). No significant differences were observed between the levels of cytotoxicity that could be induced in cells isolated between 1 and 3 days after rmGM-CSF treatment. However, 6 days after the last injection, the cytotoxicity induced by LPS was not significantly different from the level that could be induced with LPS in control peritoneal cells. Within the limited range that was tested, i.e. three and five injections of rmGM-CSF with one daily injection, no difference in the level of cytotoxicity was observed (Figure 3).

### Nitric oxide secretion

Because nitric oxide secretion has been described to play an important role in macrophage-mediated cytotoxicity, the in vitro nitric oxide secretion by peritoneal cells was measured after a 24-h incubation period with LPS. Peritoneal cells isolated from control mice did not secrete nitric oxide, but, when activated with LPS, secreted approximately 20 μM nitric oxide (Figure 4). Nitric oxide secretion by peritoneal cells isolated after rmGM-CSF treatment and stimulated in vitro with LPS was twofold higher than the nitric oxide secretion of LPS-treated control cells. The amount of nitric oxide secreted correlated with the level of cytotoxicity (compare Figures 2 and 4). The nitric oxide secretion of peritoneal cells isolated from rmGM-CSF-treated animals stimulated with LPS reached its highest level at 1 day after the last rmGM-CSF injection. In the subsequent period, the potential to secrete nitric oxide after stimulation with LPS decreased to control levels.

### Tumour cytotoxic capacity of peritoneal cells after inhibiting nitric oxide synthesis

To determine whether nitric oxide secretion by peritoneal cells was involved in tumour cell killing, nitric oxide secretion was inhibited with the nitric oxide synthase inhibitor L-NMMA. As shown in Figure 5, the level of tumour cytotoxicity of peritoneal cells is strongly inhibited by L-NMMA.

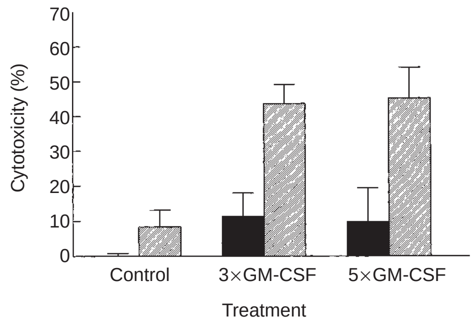

**Figure 3** rmGM-CSF was administered for 3 or 5 consecutive days with one daily injection. The control group was injected with 1 ml of 0.1% BSA in PBS for 3 consecutive days. Twenty-four hours after the last injection, peritoneal cells were harvested and the cytotoxic capacity was measured in the absence (■) or presence of 100 ng ml$^{-1}$ LPS (■) as is indicated in the Materials and methods section. Given is the percentage of cytolysis ± s.d. (*n* = 3)

### TNF-α secretion by peritoneal cells

We also studied the secretion of TNF-α by peritoneal cells. TNF-α secretion was reproducibly slightly higher in peritoneal cells isolated from rmGM-CSF-treated mice, activated in vitro with LPS, than TNF-α secretion by peritoneal cells isolated from control mice. TNF-α secretion by peritoneal cells isolated from rmGM-CSF-treated mice reached a maximal level of 21 U per $0.25 \times 10^6$ cells when activated with LPS, whereas control peritoneal cells secreted 9 U per $0.25 \times 10^6$ cells. However, TNF-α secretion was very low under both conditions and might be too low to play a significant role in the in vitro cytotoxicity towards tumour cells.

### In vitro incubation of peritoneal cells from naive mice with rmGM-CSF

Although in vivo administration of rmGM-CSF does not seem to activate peritoneal cells to tumour cytotoxicity in vivo, the cells can be stimulated with a second immunomodulator to relatively high levels of cytotoxicity. To determine whether the cytotoxic potential induced by rmGM-CSF was due to priming or to recruitment of new cells possessing a high cytotoxic potential, peritoneal cells from naive mice (resident peritoneal cells) were cultured in vitro in a 96-well plate and incubated with different concentrations of rmGM-CSF. After 24 h, the medium with rmGM-CSF was replaced by fresh medium with LPS as an activator, and the cytotoxic activity towards C26 colon carcinoma cells was measured. Figure 6 shows that rmGM-CSF is not able to prime the resident peritoneal cells in vitro because the level of LPS-induced cytotoxicity is not influenced by a preincubation with rmGM-CSF.

### DISCUSSION

To develop an optimal treatment schedule for chemoimmunotherapeutic treatment of tumour metastases confined to the peritoneal cavity, we investigated the effect of i.p. rmGM-CSF administration on the cytotoxic capacity of the peritoneal cell population of

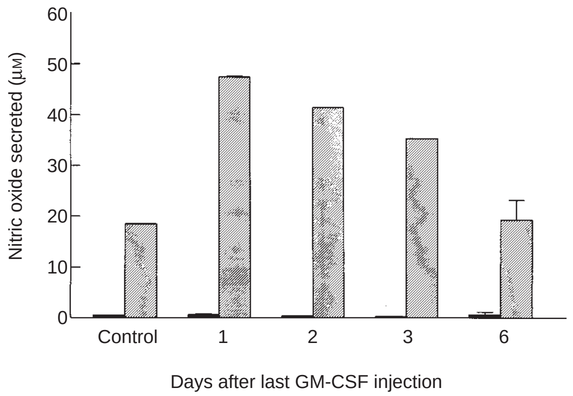

**Figure 4** Peritoneal cells were isolated from mice injected with 0.1% BSA in PBS (control group, *n* = 4) or 3000 U of rmGM-CSF (*n* = 4) for 3 consecutive days. The cells were cultured and activated in vitro with LPS (100 ng ml$^{-1}$). Nitric oxide secretion was measured 1, 2, 3 or 6 days after the last injection as described in the Materials and methods section. Nitric oxide in the culture supernatants was determined in a nitric oxide assay. Standard deviations are given when nitric oxide was determined in cultures from four separate mice. In the other cases, owing to a too low number of cells, supernatants were pooled and nitric oxide was determined in duplicate. Given is the μM nitric oxide secreted

 *© Cancer Research Campaign 1999*

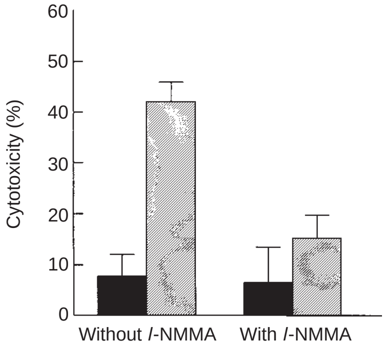

**Figure 5** Cells were harvested and pooled from two mice injected three times daily for 6 consecutive days. Cells were tested for tumour cytotoxicity as described in the Materials and methods section. The induced tumour cytotoxicity was determined in the presence or absence of 0.1 μM of the nitric oxide synthase inhibitor L-NMMA after stimulation with medium (■) or 100 ng ml$^{-1}$ (▨); all values are the mean of three wells ± s.d.

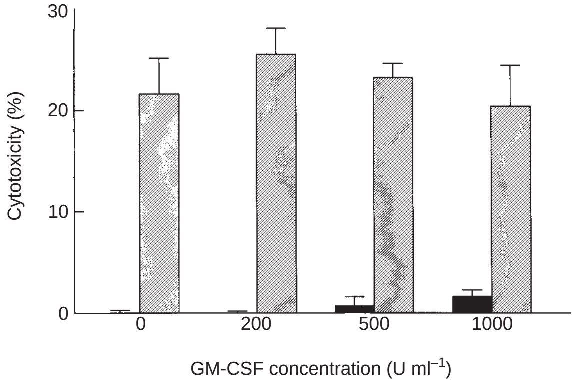

**Figure 6** Cells of three naive mice were pooled and cultured in a 96-well plate with 0.25 × 10$^{6}$ cells per well. Cells were incubated with 0, 200, 500 or 1000 U ml$^{-1}$ rmGM-CSF for 24 h. After a 24-h incubation period with rmGM-CSF, the rmGM-CSF-containing medium was replaced by fresh medium without (■) or with 100 ng ml$^{-1}$ LPS (▨) and then the cytotoxic capacity was measured as is indicated in the Materials and methods section. All values are the mean of three wells ± s.d.

mice. rmGM-CSF has been evaluated in several clinical studies for its immunomodulating properties in peritoneal dialysis patients (Selgas et al, 1996) and cancer patients (Toner et al, 1994; Aman et al, 1996; Nagler et al, 1996). In addition, GM-CSF is known to prevent toxic side-effects due to myelosuppressive therapy.

We demonstrated that the number of peritoneal cells increased with the number of rmGM-CSF injections. The influx of cells consisted mainly of newly recruited macrophages, although no increase in mitotic activity was observed. Besides an increase in the number of macrophages, the number of neutrophilic granulocytes also slightly increased. The number of peritoneal macrophages remained at an elevated level for at least 6 days whereas the increased number of neutrophilic granulocytes disappeared within 6 days after the last injection, probably because of the shorter lifetime of granulocytes compared with that of monocytes and macrophages. From this study, it is not clear whether rmGM-CSF attracts macrophages to the peritoneal cavity or leads to secretion of other cytokines or chemokines such as MIP-1α (Chen et al, 1993), MCP-1 (Reale et al, 1996; Selgas et al, 1996) or RANTES (Haelens et al, 1996) being responsible for recruitment of immune cells.

Until now, two clinical phase I studies have been described in which GM-CSF was administered i.p. (Toner et al, 1994; Selgas et al, 1996). In both studies, short-term GM-CSF administration i.p. was well tolerated, and also in humans resulted in an increase in the number of peritoneal macrophages and neutrophilic granulocytes.

We showed that cells from mice injected with rmGM-CSF were not tumour cytotoxic immediately upon isolation but have the capacity to become activated with LPS in vitro to relatively high levels, whereas cells isolated from naive or control mice had no or a significantly lower cytotoxic capacity. Macrophage tumoricidal activity was strongly inhibited by L-NMMA, indicating that nitric oxide secretion plays an important role in tumoricidal activity of peritoneal macrophages induced upon stimulation with LPS. Also, other studies showed that nitric oxide is an important mediator for tumoricidal activity of macrophages (Dileepan et al, 1995; Thomas et al, 1995).

Although the number of doses of rmGM-CSF affected the increase in cell number (Table 1), the cytotoxic capacity of the peritoneal cells was not enhanced upon repeated injections (Figure 3). Nonetheless, the total cytotoxic capacity of the peritoneal cell population was enhanced with increasing rmGM-CSF doses by the condition that more peritoneal macrophages were present in the peritoneal cavity which, as a population, have a high cytotoxic potential.

The cytotoxic potential induced by GM-CSF injection could either be due to direct priming of the peritoneal cell population by rmGM-CSF, or to recruitment of cells possessing a high cytotoxic potential from the blood circulation into the peritoneal cavity. To determine which of these pathways is operational, peritoneal cells isolated from naive mice were incubated with rmGM-CSF in vitro and the cytotoxic activity of these cells was measured in the absence and presence of LPS. These experiments showed that the resident cells were not primed by in vitro incubation with rmGM-CSF. Therefore, we tend to believe that i.p. rmGM-CSF administration causes recruitment of primed or elicited monocytes/macrophages into the peritoneal cavity. Although administration of GM-CSF has been found to exert effects also on cells of the lymphoid lineage (Steger et al, 1995; Nishijima et al, 1997), a single i.p. administration of rmGM-CSF in mice only resulted in a minor increase in the number of lymphocytes in the peritoneal cavity. Upon repeated injections, no further increase was observed. Moreover, the percentage of lymphocytes in the total peritoneal cell population was too low to play a significant role in the in vitro cytotoxicity assay.

Treatment of a peritoneal tumour with recombinant GM-CSF alone will probably not prolong the survival of tumour-bearing mice. However, GM-CSF in addition to other immunomodulating agents may lead to a significant effect on the anti-tumour activity of peritoneal cells in an immunotherapeutic treatment of peritoneal metastases. In several studies, it has been shown that antineoplastic agents, such as taxol and cisplatin, have immunostimulating effects on peritoneal macrophages (Manthey et al, 1994; Palma et al, 1994; Kirikae et al, 1996). We are presently investigating the effects of GM-CSF when combined with other immunomodulating agents in tumour-bearing mice.

Our study showed that short-term i.p. GM-CSF administration leads to an increase in peritoneal cell number by recruitment of macrophage precursor cells that have a high cytotoxic capacity. Therefore, i.p. GM-CSF administration has the potential to serve as an additional immunotherapeutic modality in the treatment of peritoneal malignancies.

© Cancer Research Campaign 1999

### ACKNOWLEDGEMENT

This study was supported by grant 94-766 from the Dutch Cancer Society.

### REFERENCES

Aliprantis AO, Diezroux G, Mulder LCF, Zychlinsky A and Lang RA (1996) Do macrophages kill through apoptosis? *Immunol Today* **17**: 573–576

Aman MJ, Stockdreher K, Thews A, Kienast K, Aulitzky WE, Farber L, Haus U, Koci B, Huber C and Peschel C (1996) Regulation of immunomodulatory functions by granulocyte–macrophage colony-stimulating factor and granulocyte colony-stimulating factor in vivo. *Ann Hematol* **73**: 231–238

Chen B, Chou T and Sensenbrenner L (1993) Induction of murine peritoneal macrophage colony-forming cells by peritoneal administration of macrophage inflammatory protein-1alfa. *Exp Hematol* **21**: 1591–1596

Daemen T, Veninga A, Roerdink FH and Scherphof GL (1986) In vitro activation of rat liver macrophages to tumoricidal activity by free or liposome-encapsulated muramyl dipeptide. *Cancer Res* **46**: 4330–4335

Daemen T, Veninga A, Roerdink FH and Scherphof GL (1989) Endocytic and tumoricidal heterogeneity of rat liver macrophage populations. *Selective Cancer Therapeutics* **5**: 157–167

Damatta RA, Araujo-Jorge T and De Souza W (1995) Subpopulations of mouse resident peritoneal macrophages fractionated on percoll gradients show differences in cell size, lectin binding and antigen expression suggestive of different stages of maturation. *Tissue Cell* **27**: 505–513

Dileepan KN, Page JC, Li, Y and Stechschulte DJ (1995) Direct activation of murine peritoneal macrophages for nitric oxide production and tumor cell killing by interferon-gamma. *J Interferon Cytokine Res* **15**: 387–394

Fidler IJ (1992) Systemic macrophage activation with liposome-entrapped immunomodulators for therapy of cancer metastasis. *Res Immunol* **143**: 199–204

Grabstein KH, Urdal DL, Tushinski RJ, Mochizuki DY, Price VL, Cantrell MA, Gillis S and Conlon PJ (1986) Induction of macrophage tumoricidal activity by granulocyte–macrophage colony-stimulating factor. *Science* **232**: 506–508

Haelens A, Wuyts A, Proost P, Struyf S, Opdenakker G and Vandamme J (1996) Leukocyte migration and activation by murine chemokines. *Immunobiology* **195**: 499–521

Inamura N, Sone S, Okubo S, Singh M and Ogura T (1990) Heterogeneity in responses of human blood monocytes to granulocyte–macrophage colony-stimulating factor. *J Leukocyte Biol* **47**: 528–534

Kirikae T, Kirikae F, Oghiso Y and Nakano M (1996) Microtubule-disrupting agents inhibit nitric oxide production in murine peritoneal macrophages stimulated with lipopolysaccharide or paclitaxel (taxol). *Infect Immunol* **64**: 3379–3384

Malik STA, Martin D, Hart I and Balkwill F (1991) Therapy of human ovarian cancer xenografts with intraperitoneal liposome encapsulated muramyl-tripeptide phosphoethanolamine (MTP-PE) and recombinant GM-CSF. *Br J Cancer* **63**: 399–403

Manthey CL, Perera P-Y, Salkowski CA and Vogel SN (1994) Taxol provides a second signal for murine macrophage tumoricidal activity. *J Immunol* **152**: 825–831

Metcalf D, Begley CG, Williamson DJ, Nice EC, DE Lamarter J, Mermod J and Thatcher D (1987) Hemopoietic responses in mice injected with purified recombinant murine GM-CSF. *Exp Hematol* **15**: 1–9

Molloy RG, Holzheimer R, Nestor M, Collins K, Mannick JA and Rodrick ML (1995) Granulocyte–macrophage colony-stimulating factor modulates immune function and improves survival after experimental thermal injury. *Br J Surg* **82**: 770–776

Nagler A, Shur I, Barak V and Fabian I (1996) Granulocyte–macrophage colony-stimulating factor dependent monocyte-mediated cytotoxicity post-autologous bone marrow transplantation. *Leukaemia Res* **20**: 637–643

Nishijima I, Nakahata T, Watanabe S, Tsuji K, Tanaka I, Hirabayashi Y, Inoue T and Karai K (1997) Hematopoietic and lymphopoietic responses in human granulocyte–macrophage colony-stimulating factor (GM-CSF) receptor transgenic mice injected with human GM-CSF. *Blood* **90**: 1031–1038

Palma JP and Aggarwal SK (1994) Cisplatin and carboplatin mediated release of cytolytic factors in murine peritoneal macrophages in vitro. *Anti-Cancer Drugs* **5**: 615–622

Plasman N and Vray B (1993) Mouse peritoneal macrophages: characterization of functional subsets following percoll density gradients. *Res Immunol* **144**: 151–163

Pojda Z, Molineux G and Dexter TM (1989) Effects of long-term in vivo treatment of mice with purified murine recombinant GM-CSF. *Exp Hematol* **17**: 1100–1104

Reale M, Frydas S, Barbacane RC, Conti P, Placido FC, Cataldo I, Anogianakis G, Dimitriadou D, Vacalis D and Trakatellis A (1996) Generation of monocyte chemotactic protein-1 (MCP-1) by rat peritoneal mast cells after LPS or TNF-alpha activation. *Int J Immunopathol Pharmacol* **9**: 109–112

Selgas R, Decastro MF, Jimenez C, Carcamo C, Contreras T, Bajo MA, Vara F and Corbi A (1996) Immunomodulation of peritoneal macrophages by granulocyte–macrophage colony-stimulating factor in humans. *Kidney Int* **50**: 2070–2078

Steger GG, Kaboo R, De Kernion JB, Figlin R and Belldegrun A (1995) The effects of granulocyte–macrophage colony-stimulating factor on tumour-infiltrating lymphocytes from renal cell carcinoma. *Br J Cancer* **72**: 101–107

Thomas C, Nijenhuis AM, Dontje B, Daemen T and Scherphof GL 1995) Tumoricidal response of liver macrophages isolated from rats bearing liver metastases of colon adenocarcinoma. *J Leukocyte Biol* **57**: 617–623

Toner GC, Gabrilove JL, Gordon M, Crown J, Jakubowski AA, Meisenberg B, Sheridan C, Boone T, Vincent ME and Markman M (1994) Phase I trial of intravenous and intraperitoneal administration of granulocyte–macrophage colony-stimulating factor. *J Immunother* **15**: 59–66

Trulson A, Nilsson S and Venge P (1996) Monocyte activation in patients with non-seminomatous germ cell tumour of the testis before and after tumour eradication. *J Clin Pathol* **49**: 381–385

Ulich TR, Del Castillo J, McNiece I, Watson L, Yin S and Andresen J (1990) Hematologic effects of recombinant murine granulocyte–macrophage colony-stimulating factor on the peripheral blood and bone marrow. *Am J Pathol* **137**: 369–376

Verstovsek S, Eppolito C, Ujhazy P, Maccubbin DL, Ehrke MJ and Mihich E (1995) Murine splenic macrophage tumoricidal activation by cytokines. *Exp Hematol* **23**: 519–528

 *© Cancer Research Campaign 1999*
